# Contamination Profiles of Perfluoroalkyl Substances (PFAS) in Groundwater in the Alluvial–Pluvial Plain of Hutuo River, China

**DOI:** 10.3390/w11112316

**Published:** 2019-11-06

**Authors:** Yan Liu, Xue Li, Xing Wang, Xiaocui Qiao, Shuran Hao, Jingrang Lu, Xiaodi Duan, Dionysios D. Dionysiou, Binghui Zheng

**Affiliations:** 1 National Engineering Laboratory for Lake Pollution Control and Ecological Restoration, Chinese Research Academy of Environmental Sciences, Beijing 10012, China; 2 State Environmental Protection Key Laboratory of Drinking Water Source Protection, Chinese Research Academy of Environmental Sciences, Beijing 100012, China; 3 U.S. Environmental Protection Agency, Office of Research and Development, Cincinnati, OH 45268, USA; 4 Environmental Engineering and Science Program, University of Cincinnati, Cincinnati, OH 45221-0012, USA

**Keywords:** perfluoroalkyl substances, groundwater, contamination profiles, alluvial–pluvial plain of Hutuo River

## Abstract

Over the past decade, concerns about perfluoroalkyl substances (PFAS) have increased rapidly among the scientific community due to their global distribution and persistence in various environmental matrices. The occurrences of 10 PFAS in groundwater in the alluvial-pluvial plain of Hutuo River (APPHR) in the North China Plain (NCP) were analyzed via UPLC-MS/MS and solid phase extraction. Total PFAS concentrations ranged from 0.56 ng/L to 13.34 ng/L, with an average value of 2.35 ng/L. Perfluorooctanoic acid (PFOA) and perfluorohexanoic acid (PFHxA) were dominant PFAS contaminants with high detection rates of 98.39% and 95.16%, respectively, and PFOA was the main pollutant with a mean concentration of 0.65 ng/L. The hydrogeological conditions have an important influence on the concentrations of PFAS in groundwater. Comparatively, the concentration of PFAS in groundwater in the study area is not very high, but it reflects that the groundwater in this region is affected by industrial sources to some extent. Local government should pay more attention on industrial pollution control and groundwater protection in this area.

## 1. Introduction

As unique water—and fat—repellent chemicals with chemical and thermal stability, perfluoroalkyl substances (PFAS) are widely applied in industrial, manufacturing, and commercial fields [1,2]. Due to extensive production and use, PFAS have spread globally in different environmental compartments, including water [2,3], sediments [4,5], biota [6], food [7,8], and human serum [9–11]. There is evidence that continued exposure above specific levels to certain PFAS may lead to adverse health effects [12]. Studies indicate that perfluorooctanoic acid (PFOA) and perfluorooctane sulfonate (PFOS) can cause reproductive and developmental, liver and kidney, and immunological effects in laboratory animals [13,14]. Both chemicals have caused tumors in animal studies. The most consistent findings from human epidemiology studies are increased cholesterol levels among exposed populations. In addition, there have been reports where PFAS are carcinogenic [15,16].

The ingestion of drinking water is a principal exposure pathway of PFAS to human beings [17–20], especially young children [21,22]. The guideline values of 3 and 0.3 μg/L for PFOA and PFOS, respectively, in drinking water can be calculated considering the European Food Safety Authority (EFSA) tolerable daily intake (1.5 μg/kg per capita and 0.15 μg/kg per capita respectively) [23,24] and applying the World Health Organization conversion rules [25]. The German Drinking Water Commission firstly set a guideline value at 0.3 μg/L for the sum of PFOA and PFOS based on the safe lifelong exposure in June 2006 [25]. An instruction value of 0.04 μg/L for PFOA in drinking water was provided in New Jersey in 2007 [26]. The United States Environmental Protection Agency (US EPA) established 200 ng/L for PFOS and 400 ng/L for PFOA as the provisional short-term health advisory level in drinking water in 2012 [27], and after the assessment of the latest peer-reviewed science in 2016, the USEPA developed the new health advisory levels at 70 ng/L for PFOS and PFOA, which provides a more effective human health protection for all Americans from a lifetime of exposure from drinking water [28].

The groundwater resources are increasingly threatened by chemical and biological pollution, which poses a significant issue, since at least half of the global population relies on groundwater. The detection of PFAS in groundwater has been reported in recent years [29–32] Nine types of PFAS were found in groundwater throughout France with a quantification frequency (QF) > 1% [27]. PFAA were recurrently detected in groundwater samples from Milan, Italy [28], with detection frequencies higher than 60%. PFOA and PFOS were collected from 20 public supply wells with a detection rate of 66% and 48%, respectively, in Massachusetts, USA [29]. PFOA was detected with concentrations from no detectable (ND) to 0.033 μg/L in 15 wells for public water supply in New Jersey, USA [30]. In China, PFAS were also found in groundwater in some areas. A total concentration of PFAS (∑PFAS) with the values of 5.3–615 ng/L in groundwater was detected in rural areas of eastern China [31]. The total per-and polyfluoroalkyl substances (∑PFASs) was up to 100 ng/L in groundwater in Tianjin City and Weifang City [32].

Located in eastern China, the North China Plain (NCP), with an area of 13.9 × 10^4^ km^2^ and a population of 107.8 million, is an important economic zone [33]. Groundwater resources are important in NCP, with groundwater supply accounting for roughly 70% of the total drinking water supply [34]. However, the groundwater in NCP suffers from contamination due to industrial and agricultural wastewater discharges [35]. Previous studies on groundwater in NCP generally focused on over-exploitation [36], geochemical identification [37], and nitrate pollution [12,38], with little information about the distribution and composition of emerging contaminants such as PFAS in the groundwater in this region.

The purpose of this study is to characterize the pollution profiles of PFAS in groundwater within NCP, including their occurrence, concentration levels, and spatial distribution. In addition, this study examines the effect of hydrogeological conditions on the contamination profiles of PFAS in groundwater and explores the potential sources of PFAS in groundwater in NCP.

## 2. Materials and Methods

### 2.1. Study Area

The Hutuo River Plain in the west of the NCP is located in the piedmont recharge area, and the quality of the groundwater in the area is critical to the entire central region of the NCP. Therefore, it is important and meaningful to do pilot studies and assessments on the contaminated status for investigating groundwater quality [39]. The area has a continental monsoonal climate with an average precipitation of 534 mm per year, and around 70% of the precipitation takes place from July to September. The recharge channels of groundwater in the study area mainly include precipitation, river infiltration, and irrigation return.

The buried depth of the groundwater and lithologic properties of aquifers have obvious zonation patterns. Thus, the study area was divided into four groundwater units: Fissure and pore water unit in the valley in Gangnan Reservoir and Huangbizhuang Reservoir (G1), Pore water unit in the top alluvial-pluvial plain of Hutuo River (APPHR) (G2), Shallow pore water unit in the middle APPHR (G3), and a Deep pore water unit in the middle APPHR (G4) [40]. The thickness of aquifer in G1 ranges between 10 and 20 m, with buried depths of 2–20 m, and the aquifer media consists of quaternary unconsolidated sediments and fracture gneiss and marble rock. The aquifer in G2 is mainly composed of gravel and sand gravel, and the permeability is high with a permeability coefficient of 300–400 m/day. The thickness of aquifer in G2 ranged between 20 and 35 m. The aquifer in G3 is formed by sand–gravel, coarse sand with gravel, and medium-coarse sand, with good water conduction and water production performance, and the permeability coefficient is 100–200 m/day. The buried depth of the aquifer in G3 is 80–100 m with a thickness of 25–60 m. The aquifer in G4, underlying the aquifer in G3, consists of sand–cobble and coarse sand with gravel. The buried depth of aquifer in G4 is 125–238 m with a thickness of 110–140 m [41].

### 2.2. Sampling

**Figure 1.**
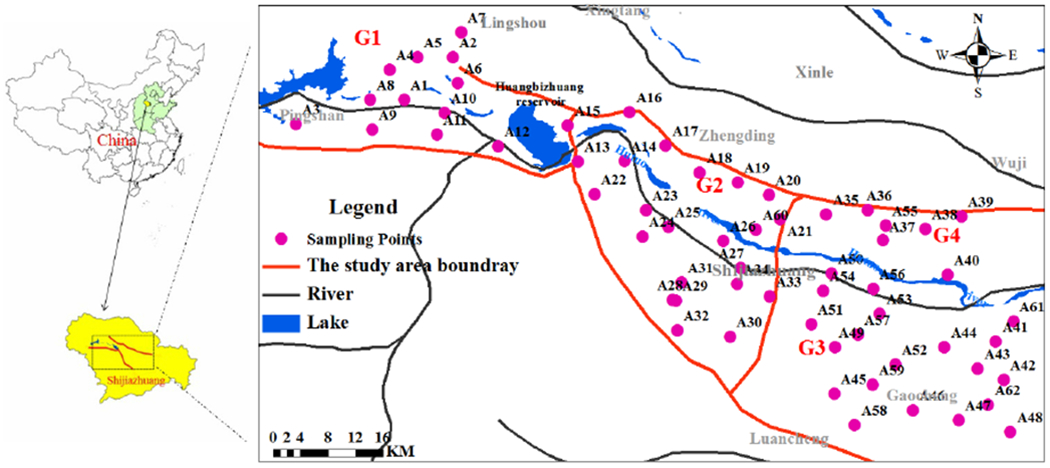
Sampling locations in the alluvial-pluvial plain of Hutuo River (APPHR) in Shijiazhuang City (G3 and G4 share the same area in the horizontal direction, but are located in different depths in the vertical direction). G1: Fissure and pore water unit in the valley in Gangnan Reservoir and Huangbizhuang Reservoir, G2: Pore water unit in the top APPHR, G3: Shallow pore water unit in the middle APPHR, G4: a Deep pore water unit in the middle APPHR.

A total of 62 groundwater samples were collected from November to December in 2014. The sampling information of groundwater in four units for the sampling sites is shown in Figure 1.

All the groundwater samples from each well were obtained after 20 min of pumping, generally until the pH, temperature (T), electrical conductivity (EC), and oxidation–reduction potential (ORP) in the flowing water remained stable. The pH, T, EC, DO, and ORP were measured by a multi-parameter portable meter (Multi 3510 ISS, WTW, Munich, Germany) on site. All samples were contained in 4-L polypropylene (PP) bottles (Nalgene, ThermoFisher, Shanghai, China) and stored at 4 °C after sampling for subsequent laboratory analysis.

### 2.3. Chemicals and Reagents

Perfluorohexanoic acid (PFHxA, 97%), perfluorobutanoic acid (PFBA, 95%), perfluorobutane sulfonate (PFBS, 98%), perfluoropentanoic acid (PFPeA, 97%), perfluorohexane sulfonate (PFHxS, 99%), perfluoroheptanoic acid (PFHpA, 99%), and PFOS (99%) were purchased from Sigma-Aldrich Chemical (St Louis, MO, USA). PFOA was purchased from Fisher Chemical (Hanover park, IL, USA). Perfluorononanoic acid (PFNA, >98%), perfluorodecanoic acid (PFDA, >98%), perfluoro-(^13^C4)-octanoic acid (^13^C4-PFOA, 99%), and perfluoro-(^13^C4)-octane sulfonic acid (^13^C4-PFOS, 99%) were purchased from Wellington laboratories (Guelph, ON, Canada).

Methanol of high-performance liquid chromatography (HPLC) grade and ammonium acetate of analytical grade were purchased from Fisher Chemical Co. (Hanover park, IL, USA).

### 2.4. Extraction and Analysis

The water sample extraction procedure was adapted from the previous reports [41,42] with some modifications. The Oasis HLB extraction cartridges (0.5 g, 6 mL) (Waters Corp., Milford, MA, USA) were preconditioned by passing 5 mL of methanol and 5 mL of nanopure water successively with a rate of 2 drops per second. Next, 2 ng of the internal standard (MPFOS and MPFOA) was added to 1 L of samples, and then the mixture was loaded onto the cartridge with a rate of 1 drop per second. Then, 5 mL of 20% methanol was used to wash the cartridge, and finally, the target fraction was enriched in a 5-mL PP centrifuge tube using 5 mL of methanol at a rate of 2 drops per second. The eluate was concentrated to 0.25 mL under a nitrogen stream (14165-C, Organmation, Berlin, MA, USA), and diluted using 2 mL of nanopure water. After a brief vortex time, the tube was centrifuged at 12,000 rpm for 5 min (1–14, Sigma, Milwaukee, WI, USA). Before UPLC MS/MS measurement, the eluent was evaporated to 0.5 mL by high-purity nitrogen and passed through a 0.22-mm organic phase nylon syringe filter (ANPEL Laboratory Technologies (Shanghai) Inc., Shanghai, China). Analysis of PFAS in samples was performed in ultraperformance liquid chromatography (UPLC, Waters Corp., Milford, MA, USA) interfaced with a Quattro Premier XE tandem quadrupole mass spectrometer (MS/MS, Waters Corp., Milford, MA, USA), and operated under electrospray negative ionization (ESI) mode. The separation was carried out with an ACQUITY UPLC-TM BEH C18 column (2.1 mm × 50 mm, 1.7 μm, Waters Corp., Milford, MA, USA). Milli-Q water containing 10 mmol L^−1^ of ammonium acetate was used as the aqueous phase (A), and the organic phase (B) was 10 mmol L^−1^ of ammonium acetate in 8:2 (*v/v*) methanol/acetonitrile. The gradient started from 50% A, decreased to 0% A at 7 min, increased to 50% A at 7.5 min, and then was kept the same to 9.0 min. The injection volume was 10 μL with a flow rate of 0.3 mL/min, and the column temperature was held at 35 °C. Multiple reactions monitoring (MRM) mode was applied in the MS/MS analysis. The temperature of desolvation gas was 450 °C, and the ion spray voltage was 0.44 kV. The cone voltages and the collision energies for 10 PFAS, MPFOA, and MPFOS were provided in the Supporting Information.

### 2.5. Quality Assurance and Control

Quality assurance and control procedures were followed during the sampling, extraction, and analysis. MPFOS and MPFOA were used as standards for internal calibration. Seven-point calibrations with concentrations of 0.025, 0.05, 0.1, 0.2, 0.5, 1, and 2 ng/mL were prepared in methanol, and the determination coefficients of the calibration curves were above 0.99. Blanks and control samples were run every 6 samples to check for precision and accuracy of the recovery. The limit of detection (LOD) was determined 3 times with a signal-to-noise (S/N) ratio, while the limit of quantification (LOQ) was determined with a S/N ratio of 10:1. The LOD of PFAS was 0.01–0.14 ng/L. The recoveries of 10 PFAS ranged from 87% to 101.7%, and their relative standard deviation (RSD) values were under 10% (n = 6, listed in Table S1).

### 2.6. Statistical Analysis

The Spearman’s rank correlation was used to discuss the possible sources of pollution. The data was normalized before principal component analysis. Statistical evaluation analysis was conducted using the software SPSS 16.0 (SPSS Inc., Chicago, IL, USA). Values lower than LOQ were reported as half of the LOQ, and those lower than LOD were reported as ND. A value of “zero” was assigned for the statistical purpose.

## 3. Results and Discussion

### 3.1. Basic Properties of Groundwater

The T, pH, DO, and ORP of the samples are shown in Table S2. The T was in the range of 6.7–17.6 °C, and the mean value of 14.91 °C. The pH ranged between 6.82 and 8.28, with a mean value of 7.53, indicating that the pH of the samples of the study area was in a neutral and slightly alkaline range. The DO concentration of the samples varied in the range of 0.03–8.90 mg/L.

### 3.2. Occurrence of PFAS in Groundwater

**Figure 2.**
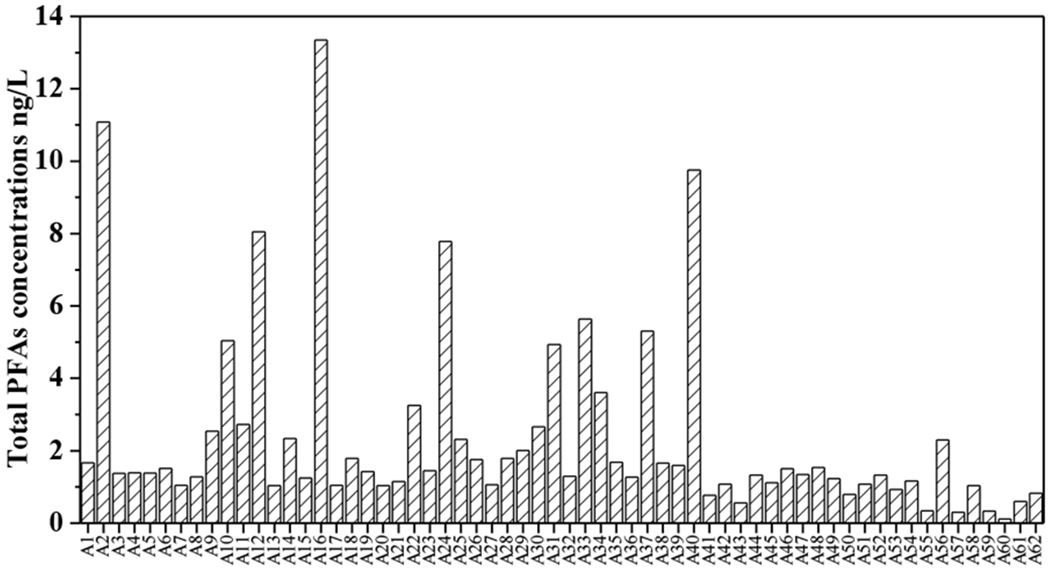
Total concentration of the 10 studied PFAS compounds (ng/L) in 62 groundwater samples in the APPHR in Shijiazhuang City.

The total concentrations of the 10 PFAS (∑PFAS) ranged from 0.11 ng/L to 13.34 ng/L. The mean value and median value were 2.35 ng/L and 1.39 ng/L, respectively. The highest ∑PFAS was observed in A16 in G2, followed by A2 with the ∑PFAS of 11.08 ng/L in G1 (Figure 2).

All 10 PFAS could be detected in 21% of all groundwater samples. PFDA was detected in 98.39% of samples, followed by PFOA with the detection frequency of 95.16%. PFBA, PFHxA, and PFNA were also detected at a high detection frequency (90.32%). The lowest detection frequency was obtained for PFOS (48.39%).

**Figure 3.**
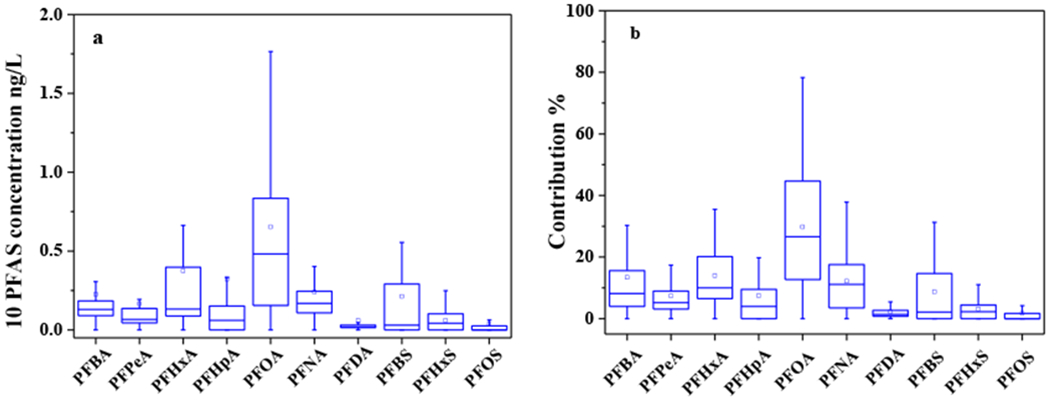
Concentrations of PFAS (**a**) and their contributions (**b**) in groundwater samples from APPHR in Shijiazhuang City.

The concentrations of 10 PFAS and the contribution of PFAS to the ∑PFAS in groundwater samples are summarized in Figure 3. The mean concentration of PFOA was 0.65 ng/L with the range from ND to 4.27 ng/L, followed by PFHxA and PFHpA with mean concentrations of 0.37 ng/L and 0.32 ng/L, respectively. In addition, the most prominent contribution was obtained from PFOA (30.07%), and the second highest contribution was obtained from PFHxA (13.87%) followed by PFBA (13.48%). The individual percentage contributions of PFOS, PFHxS, and PFDA were less than 4%, and the lowest contribution was that of PFOS (1.67%). Accordingly, PFOA and PFHxA were predominant PFAS in the groundwater samples in the investigated area with a high detection frequency and high concentration.

In China, PFAS were also found in groundwater in some areas. A total concentration of PFAS (∑PFAS) with the values of 5.3–615 ng/L in groundwater was detected in rural areas of eastern China [31]. Total per-and polyfluoroalkyl substances (∑PFASs) was up to 100 ng/L in groundwater, which was possibly due to severe point sources in Tianjin City and Weifang City [32]. The mean value of ∑PFAS was also analyzed. The mean of ∑PFAS (2.35 ng/L) in the study was compared with the results from the literature. The mean of ∑PFAS in the study area was higher than that detected in groundwater in Tai’an, China (1.68 ng/L), but relatively lower than that found in groundwater in Changshu China (269.1 ng/L), Yangzhou, China (8.5 ng/L), and Yancheng, China (3.57 ng/L) in 2014 [43]. In Sweden, the mean groundwater (n = 161) concentration of ∑26PFASs was 49 ng/L (median 0.04 ng/L, 2015) [44]. In French Overseas Territories (French Guiana, Guadeloupe, Martinique, Mayotte and Reunion, 2012), the PFAS concentration ranged from LOD to 638 ng/L (median = 0.56 ng L^−1^) in groundwater (n = 80) [45].

### 3.3. Effects of Groundwater Hydrological Conditions on PFAS Distribution

**Figure 4.**
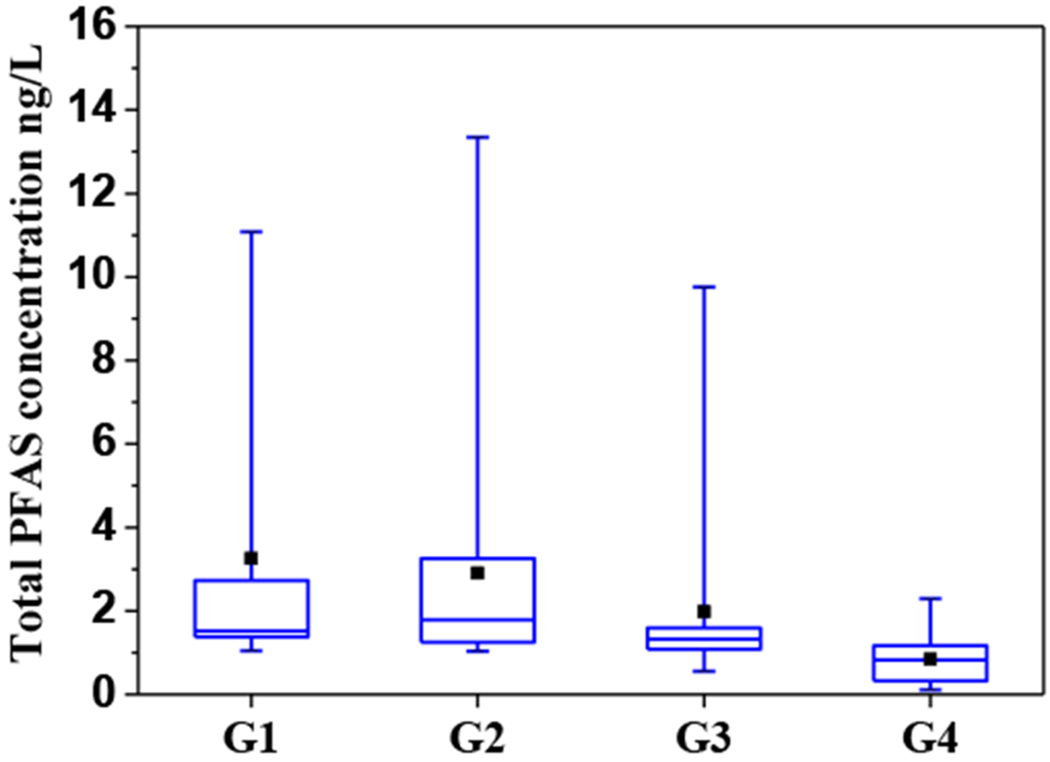
Total concentrations (ng/L) of PFAS in groundwater samples in four regions from the APPHR in Shijiazhuang City.

The PFAS concentrations in water samples from four regions are summarized in Figure 4. The mean of ∑PFAS in the G1, G2, G3, and G4 are 3.26 ng/L, 2.91 ng/L, 1.97 ng/L, and 0.842 ng/L, respectively, which shows a decreasing trend.

**Figure 5.**
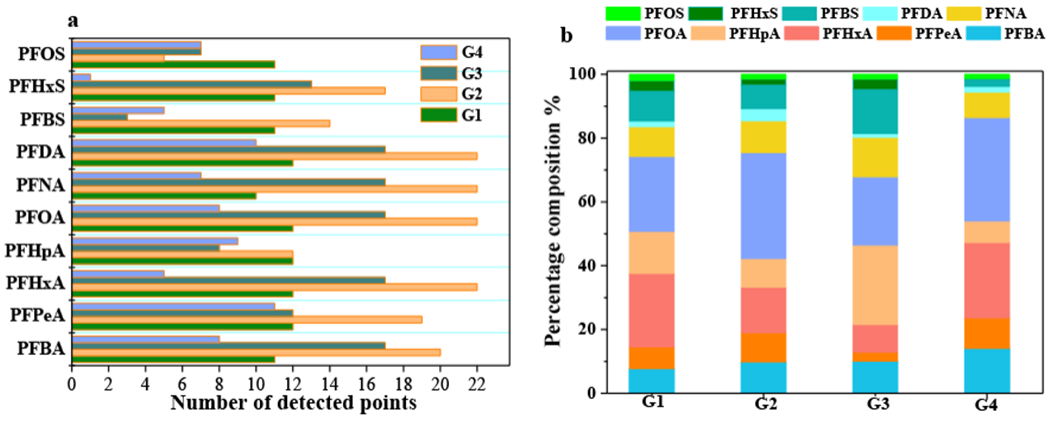
Number of detected points (**a**) and percentages of **10** PFAS in groundwater samples (**b**) in four regions from the APPHR in Shijiazhuang City.

The composition and detection categories of PFAS in the four units were studied. As shown in Figure 5, all 10 PFAS could be detected in the four units. In G1, the detection frequencies of PFOA, PFPeA, PFHxA, PFHpA, and PFBS were 100%. The PFNA was found at a detection frequency of 83.3%. The detection frequencies of the other PFAS were 92%. In G2, the detection frequencies of PFNA, PFHxA, PFOA, and PFDA were 100%, while the PFOS had the lowest detection frequency of only 22.73%. In G3, the detection frequencies of PFBA, PFHxA, PFOA, PFNA, and PFDA were 100%, while the lowest detection frequency was obtained for PFBS, which was only 17.65%. In G4, the detection frequencies of PFPeA PFDA, and PFHxS were 100%, 64%, and 9%, respectively.

**Figure 6.**
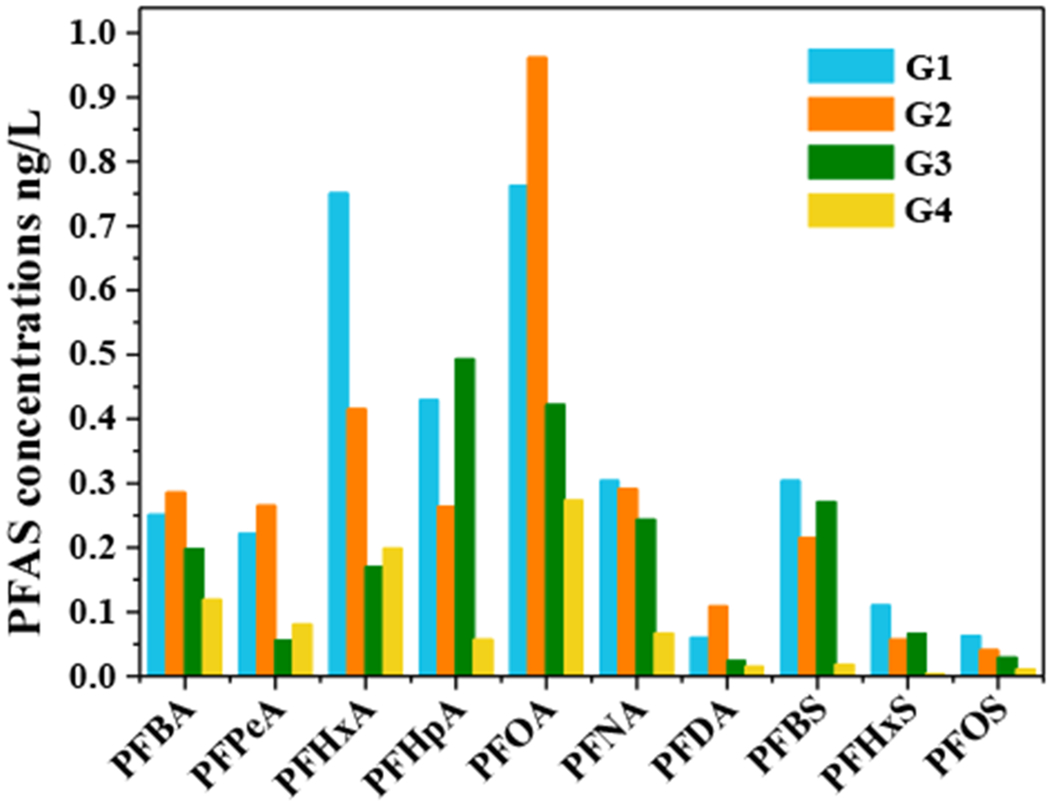
Concentrations of 10 PFAS in groundwater samples of four regions from the APPHR.

The average concentrations of 10 PFAS in groundwater samples from the four units in the studied area are shown in Figure 6. PFOA was found in G1, G2, and G4, with the highest average concentration of 0.76 ng/L, 0.96 ng/L, and 0.27 ng/L, respectively. However, the PFHpA was the prominent PFAS in G3 with the highest average concentration of 0.49 ng/L. The PFAS with the lowest concentration had great differences among the four units. PFDA was found in G1 and G3 with the lowest average concentrations of 0.06 ng/L and 0.024 ng/L, respectively. In G2, the PFOS had the lowest average concentration of 0.041 ng/L. In G4, the average detectable concentration of PFHxS at each point was lower than the LOD.

The occurrence and migration of PFAS were likely affected by the local hydrogeological environment. As described in Section 2.1, rocks in four units have different porosity and permeability characteristics. The seepage zone of G1 and G2 reveals coarse lithology and good permeability, thus potentially increasing the susceptibility and resulting in the easy permeation of surface PFAS to the aquifer. Therefore, PFAS with high concentrations were detected in the above two units. The particle size in the aquifer in G3 is smaller than that in G1 and G2; thus, low permeability and movement of contaminants can be foreseeable. Additionally, the increase of the thickness in the intermediate layer and the buried depth of the aquifer in G3-made surface water is relatively difficult to get into underground. Therefore, the lower ∑PFAS was found in G3. G4 is below G3, and the thickness of the aquifer in G4 is increased, as well as the buried depth. On the average, the porosity and permeability of rocks decrease as their depth below land surface increases; thus, lower sensitivity and susceptibility are found in the aquifer layer in G4. Such hydrogeological features could be the explanation for the lowest ∑PFAS in G4 among the four units.

### 3.4. Compositional Profiles of PFAS and Source Identification

Some information on pollution sources may be obtained from the composition of PFAS in samples to some extent [46]. The results of groundwater principal component analysis indicated that two principal components were selected by system in default (Table S4). The two principal components accumulatively explained 75.26% of the total variances. Factor analysis suggested that the first main component was the category of “short-chained PFAS (C4-C7)”: PFBA, PFPeA, PFHxA, PFHpA, PFBS, and PFHxS, explaining 44.42% of the total variances, and the second main component was the category of “long-chained PFAS (C8–C14)”: PFOA, PFNA, PFDA, PFOS, explaining 20.84% of the total variances. The short-chained and long-chained PFAS in the groundwater in the study area could possibly come from the same or similar pollution sources.

The ratio of PFHpA to PFOA has been employed to discriminate between point and diffuse sources of PFAS to surface water, and it might be inferred that atmospheric sources associated with urban areas did not make a big contribution if the PFHpA: PFOA ratio (0.354) was less than one [47]. The PFHpA:PFOA ratio was in the range of 0–0.95 in the study area, which suggested that atmospheric deposition was not the main PFAS source in this watershed. Spearman rank correlations among the 10 studied PFAS in groundwater were examined, and results are presented in Table 1. PFOA had a strong correlation with PFNA (r = 0.512, p = 0.01). It was reported that PFOA and PFNA could probably be produced by the biodegradation of the same precursors such as telomer alcohols [46], so a possible source of PFOA and PFNA could be the biodegradation of their precursors. In additional, significant correlations between PFHxA and PFPeA (r = 0.603, p = 0.01), PFHxA and PFOA (r = 0.525, p = 0.01), PFHxA and PFNA (r = 0.491, p = 0.01), and PFHxA and PFDA (r = 0.510, p = 0.01) were also found (Table 1). PFHxA is used as new material in the manufacturing industry in China [48] therefore, the PFHxA as well as PFPeA, PFOA, PFNA, and PFDA probably all came from industrial sources. Additionally, PFNA was found to have a close relationship with PFDA. Sun et al. showed that fluorotelomer alcohols (FTOH) might yield even—and odd—chain-length perfluoroalkyl carboxylic acids (PFCAs) such as PFNA and PFDA [49].

## 4. Conclusions

PFAS are widely present in the groundwater of APPHR in NCP in China, and hydrogeological conditions show certain effects on the concentration levels of PFCs in different groundwater areas.

1. The ∑PFAS ranged from 0.56 to 13.34 ng/L, and the PFOA and PFHxA were dominant PFAS contaminants with high detection frequencies of 98.39% and 95.16%, respectively. Generally, the concentrations of PFAS in groundwater in NCP are not very high compared to previous reports in other areas in China [48]. Compared to other regions worldwide, the PFAS contamination levels in this study were in the range of slightly to moderately impacted [31,45].
2. The distribution of PFAS in the study area was affected by the hydrogeological conditions. The average concentrations of PFAS were the highest in the Fissure and pore water unit in the valley in Gangnan Reservoir and Huangbizhuang Reservoir (G1), followed by Pore water unit in the top APPHR (G2), Shallow pore water unit in the middle APPHR (G3), and Deep pore water unit in the middle APPHR (G4).
3. Principal component analysis suggested that the short-chained PFAS (C4–C7) had the same pollution sources as the long-chained PFAS (C8–C10), and removing long-chain PFAS from water during the infiltration process is adsorbed by soil and rock. Spearman correlation analysis further indicated that the precursors’ biodegradation might have an important contribution to the presence of PFOA and PFNA.

## Supplementary Material

Supplemental

## Figures and Tables

**Table 1.** Spearman correlation coefficients (two-tailed) for individual PFAS concentrations in groundwater of APPHR (n = 62). PFBA: perfluorobutanoic acid, PFPeA; perfluoropentanoic acid, PFHxA: perfluorohexanoic acid, PFHpA: perfluoroheptanoic acid, PFOA: perfluorooctanoic acid, PFNA: perfluorononanoic acid, PFDA: perfluorodecanoic acid, PFBS: perfluorobutane sulfonate, PFHxS: perfluorohexane sulfonate, PFOS: perfluorooctane sulfonate.

	PFBA	PFPeA	PFHxA	PFHpA	PFOA	PFNA	PFDA	PFBS	PFHxS	PFOS
PFBA	1									
PFPeA	0.028	1								
PFHxA	0.145	0.603 **	1							
PFHpA	−0.051	0.291 *	0.414 **	1						
PFOA	0.106	0.475 **	0.525 **	0.354 *	1					
PFNA	0.267 *	0.162	0.491 **	0.382 **	0.512 **	1				
PFDA	−0.023	0.215	0.510 **	0.275 *	0.189	0.669 **	1			
PFBS	0.051	0.024	0.100	0.002	0.007	0.070	0.126	1		
PFHxS	0.204	0.178	0.371	0.223	0.161	0.362	0.130	0.418	1	
PFOS	−0.021	0.163	0.441	0.523	0.095	0.613	0.818	0.075	0.382	1

Notes:

** Correlation is significant at the 0.01 level (2-tailed).

* Correlation is significant at the 0.05 level (2-tailed).
