# Supplementary material for: Contamination Profiles of Perfluoroalkyl Substances (PFAS) in Groundwater in the Alluvial–Pluvial Plain of Hutuo River, China
Source: Water (Basel). Author manuscript; Available in PMC 2020 Nov 6. (PMC6997942; doi:10.3390/w11112316)
Supplement: Supplemental [file NIHMS1547631-supplement-Supplemental.docx]

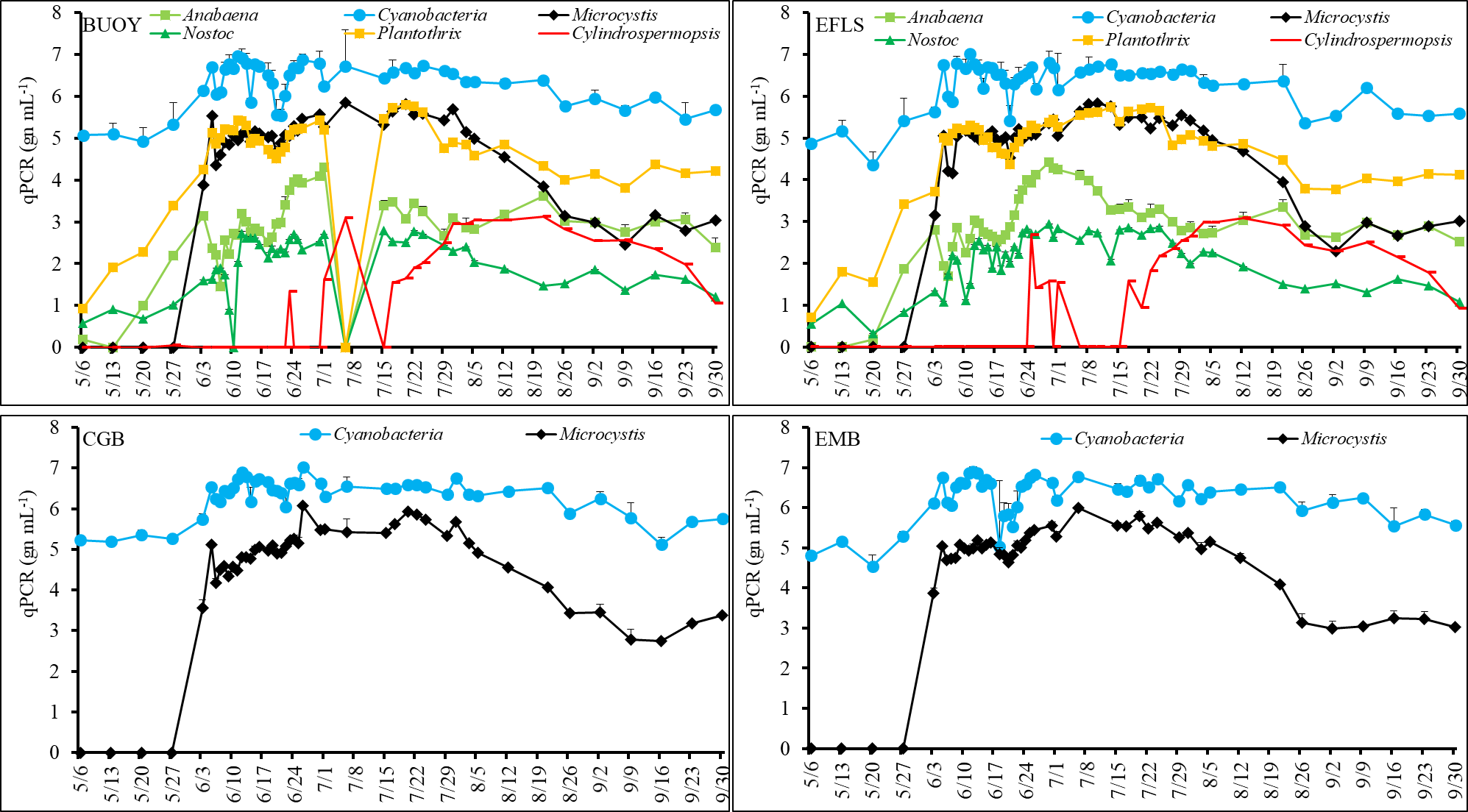


Fig. S1. The qPCR of cyanobacteria, *Microcystis*, *Anabaena*, *Planktothrix, Cylindrospermopsis* and *Nostoc* in the surface water sites


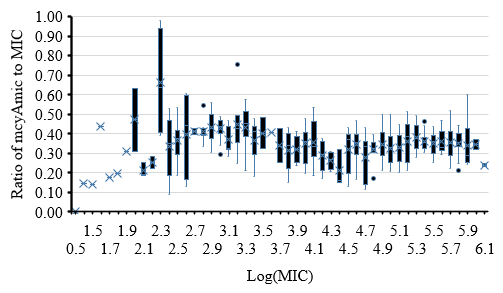


Fig. S2. Correlation of ratio of microcystin producing subpopulations (mcyAmic) to *Microcystis* (MIC): the ratios trended lower with increasing of *Microcystis* spp., but the subpopulations kept a pretty constant ratio to *Microcystis* population.


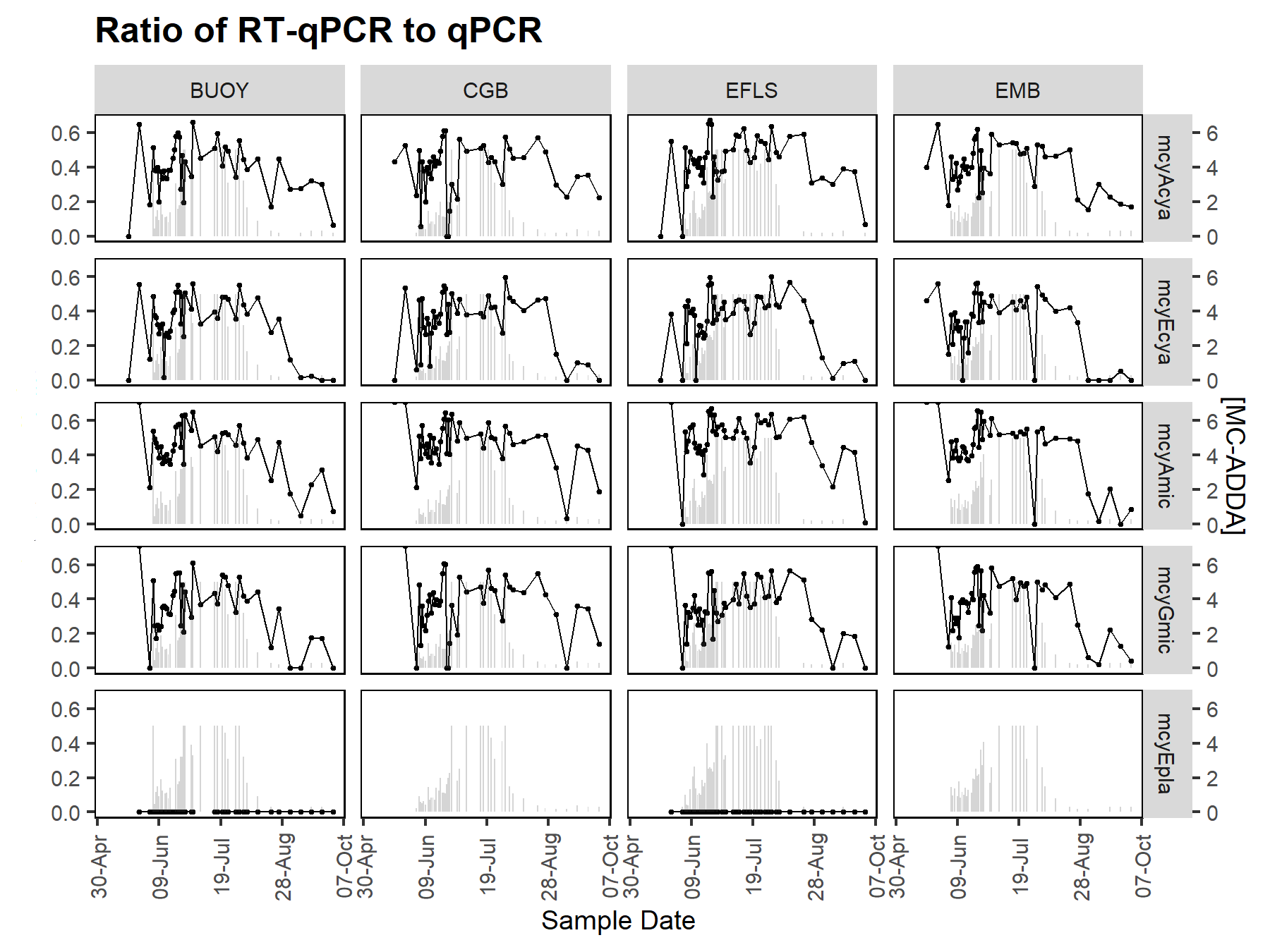


Fig. S3. Ratios of RT-qPCR to qPCR (y-axis), showing mcyA, E and G gene transcripts against extant copy numbers

Table S1 USEPA Harsha Lake sampling sites in 2015.

| Site ID | Site Name | latitude | longitude |
| --- | --- | --- | --- |
| EMB | East Fork Lake Main Beach west of narrows | 39.02 | -84.1311 |
| BUOY | Harsha Buoy | 39.032506 | -84.137661 |
| CGB | Lake Harsha Campground Beach | 39.022506 | -84.094618 |
| EFLS | East Fork Lake at DWTP intake Surface | 39.0367 | -84.1381 |
| EFLD | East Fork Lake at DWTP intake Depth | 39.0367 | -84.1381 |

| Table S2 Test of RNA and mRNA recovery by RT-qPCR from preserved field samples | | | | | | |  |  |  |  |
| --- | --- | --- | --- | --- | --- | --- | --- | --- | --- | --- |
|  |  | 1 freeze-thaw cycle | |  |  |  | 2 freeze-thaw cycles | |  |  |
| Assay | Time | Mean  (log gn L^-1^) | StDev   (log gn L^-1^) | Deduction^1^   (log gn L^-1^) | P values  of t-test | Time | Mean   (log gn L^-1^) | StDev  (log gn L^-1^) | Deduction   (log gn L^-1^) | T-test |
| MIC | 1 day | 10.338 | 0.121 |  |  | 1 day | 10.638 | 0.122 |  |  |
|  | 1 wk^2^ | 9.769 | 0.093 | 0.569 | 0.006 | 1 wk | 10.020 | 0.048 | 0.618 | 0.005 |
|  | 2 wk | 10.389 | 0.214 | 0.050 | 0.743 | 2 wk | 10.389 | 0.214 | 0.250 | 0.200 |
|  | 3 wk | 10.051 | 0.077 | 0.287 | 0.047 |  |  |  |  |  |
| mcyAms | 1 day | 4.356 | 0.427 |  |  | 1 day | 4.631 | 0.388 |  |  |
|  | 1 wk | 3.536 | 0.009 | 0.820 | 0.083 | 1 wk | 3.680 | 0.248 | 0.951 | 0.093 |
|  | 2 wk | 3.887 | 0.337 | 0.469 | 0.340 | 2 wk | 3.887 | 0.337 | 0.744 | 0.174 |
|  | 3 mk | 3.785 | 0.105 | 0.571 | 0.175 |  |  |  |  |  |

1. Deduction of log gn L^-1^ from 1-day to 1, 2 or 3 week. 2. Significant degradation might be caused by operation during process for 1-week samples.

| Oligo name | Sequences (5'-3') | Tm (°C) | Target | bp | Limit of detection (gn rx^-1^) | Reference |
| --- | --- | --- | --- | --- | --- | --- |
| CYAf | GGGGAATYTTCCGCAATGGG | 60 | Cyanobacteria | 423 | 1 | Nubel et al. 1997 |
| CYAr | GACTACAGGGGTATCTAATCCCWT T |  |  |  |  |  |
| MICf | ATGTGCCGCGAGGTGAAACCTAAT | 60 | 16S rRNA in *Microcystis* | 248 | 1 | Neilan et al. 1997 |
| MICr | TTACAATCCAAAGACCTTCCTCCC |  |  |  |  |  |
| mcyEcyaf | TTTGGGGTTAACTTTTTTGGGCATAGTC | 56 | *mcyE* or *ndaF* in Cyanobacteria | 470 | 10 | Jungblut and Neilan, 2006 |
| mcyEcyar | AATTCTTGAGGCTGTAAATCGGGTTT |  |  |  |  |  |
| mcyAcyaf | AAAAGTGTTTTATTAGCGGCTCAT | 56 | *mcyA* in Cyanobacteria | 302 | 10 | Hisbergues 2003 |
| mcyAcyar | ATCCAGCAGTTGAGCAAGC |  |  |  |  |  |
| mcyAmsf | ATCCAGCAGTTGAGCAA | 60 | *mcyA* in *Microcystis* | 171 | 10 | Furukawa et al., 2006 |
| mcyAmsr | GCCGATGTTTGGCTGTAAAT |  |  |  |  |  |
| mcyGmicf | CAACCCAACAGGTTCTTAAAGC | 60 | *mcyG* in *Microcystis* | 244 | 10 | Ngwa, 2012 |
| mcyGmicr | TGAGGCAAGGTTTCCTCTTG |  |  |  |  |  |
| Cy_rpo12 | GGCATTCCTAGTTATATTGCCATACTA | 56 | *rpoC* in *C. raciborskii* | 308 |  | Wilson et al., 2000 |
| Cy_rpo14 | GCCCGTTTTTGTCCCTTTGCTGC |  |  |  |  |  |
| NTSf | TGTGATGCAAATCTMA | 56 | 16S rRNA in *Nostoc* | 200 |  | Moffitt 2001, Neilan et al., 1997 |
| 1492R | TACGGCTACCTTGTTACGAC |  |  |  |  |  |
| PLAf | AACCCATAGGGAGATAACTC | 60 | phycocyanin in *Planktothrix* | 131 |  | Baxa et al. 2010 |
| PLAr | GCTTTGGCTTGACGGAAACG |  |  |  |  |  |
| mcyEplaf | GAAATTTGTGTAGAAGGTGC | 60 | *mcyE* in *Planktothrix* | 249 |  | Vaitomaa et al., 2003 |
| mcyEplar | CTCAATCTGAGGATAACGAT |  |  |  |  | Rantala 2006 |

Table S3. Primer used for SYBR Green based qPCR and RT-qPCR assays

Table S4. Specificity test for qPCR assays

| **Order/family** | **Genus** | **Species (** | mcyEcya | mcyAcya | mcyAmic | mcyGmic | MIC | CYA | NS2f | PlA | mcyEpla |
| --- | --- | --- | --- | --- | --- | --- | --- | --- | --- | --- | --- |
| *Chroococcales /* | Aphanothece | *Ap. Paralelliformis* | - | - | - | - | - | + | - | - | - |
| *Aphanothecoidae* |  | *Ap.* Sp*.* (43922) | - | - | - | - | - | + | - | - | - |
|  | Cyanodictyon | *Cy. planctonicum* | - | - | - | - | - | + | - | - | - |
| *Chroococcales /* | Gomphosphaeria | [Gomphosphaeria sp.](http://www.algaebase.org/search/?genus=Gomphosphaeria) | - | - | - | - | - | + | - | - | - |
| [Gomphosphaeriaceae](http://www.algaebase.org/search/genus/detail/?genus_id=r9463a62b9ddbbc49) | *Lemmermanniella* | *Lemmermanniella sp.* | - | - | - | - | - | + | - | - | - |
|  | *Chroococcus* |  | - | - | - | - | - | + | - | - | - |
|  | Cyanobacterium |  | - | - | - | - | - | + | - | - | - |
|  | Geminocystis |  | - | - | - | - | - | + | - | - | - |
|  | Microcystis | MA2385-5 | + | + | + | + | + | + | - | - | - |
|  |  | MA2388-1 | + | + | + | + | + | + | - | - | - |
|  |  | MA2386 4-1-14 | - | - | - | - | + | + | - | - | - |
|  |  | MA2385 4-1-14 | + | + | + | + | + | + | - | - | - |
|  |  | MA2388 4-1-14 | + | + | + | + | + | + | - | - | - |
|  |  | WA2385-10 | + | + | + | + | + | + | - | - | - |
|  |  | 2688 4-1-14 | - | - | - | - | + | + | - | - | - |
|  |  | 2385 1-23-13 | - | - | - | - | + | + | - | - | - |
|  |  | NIES 843 | + | + | + | + | + | + | - | - | - |
|  |  | 2385-15 1-15-15 | + | + | + | + | + | + | - | - | - |
|  |  | GLSM UC 3A | - | - | - | + | + | + | - | - | - |
|  |  | 2385-15 5-9-15 | + | + | + | + | + | + | - | - | - |
|  |  | 2386-1 | - | - | - | - | + | + | - | - | - |
|  |  | 2386-2 | + | + | + | + | + | + | - | - | - |
|  |  | 2385 9-26-13 | + | + | + | + | + | + | - | - | - |
|  |  | 2388-3 | + | + | + | + | + | + | - | + | - |
|  |  | GLSM UC 4A | + | + | + | + | + | + | - | - | - |
|  |  | Anabaena | - | - | - | - | - | + | + | - | - |
|  |  | Nostac | - | - | - | - | - | + | + | - | - |
|  |  | Oscillatoria | - | - | - | - | - | + | - | - | - |
|  |  | Lyngbya | - | - | - | - | - | + | + | - | - |
|  |  | AFA | - | - | - | - | - | + | + | - | - |
|  |  | MA | + | + | + | + | + | + | - | - | - |
|  |  | *Scendesmus* | - | - | - | - | - | - | - | - | - |
|  |  | Extract blank 1 | - | - | - | - | - | - | - | - | - |
|  |  | Extract blank 2 | - | - | - | - | - | - | - | - | - |
|  |  | NTC | - | - | - | - | - | - | - | - | - |
|  |  | NTC | - | - | - | - | - | - | - | - | - |

Table S5. LC/MS/MS operating conditions for MC congener analysis

|  | **MS/MS Conditions** | | | | **LC Conditions** | | |
| --- | --- | --- | --- | --- | --- | --- | --- |
| **Analyte** | **MS/MS Transition** | **Cone Voltage (V)** | **Collision Energy (eV)** | **Reporting Limit (µg/L)** | **Time (min)** | **% 20 mM NH_4_HCO_2_** | **% Methanol** |
| Nodularin | 825.4 > 134.9 | 45 | 55 | 0.037 | Initial | 90 | 10 |
| MC-YR | 523.4 > 134.9 | 20 | 20 | 0.15 | 2.0 | 90 | 10 |
| MC-HtyR | 1059.6 > 134.9 | 60 | 75 | 0.15 | 16.0 | 20 | 80 |
| MC-RR | 519.9 > 134.9 | 35 | 30 | 0.025 | 16.1 | 10 | 90 |
| 3-desmethylated-MC-RR | 512.9 > 134.9 | 40 | 30 | 0.075 | 22.0 | 10 | 90 |
| MC-LR | 995.5 > 134.9 | 60 | 70 | 0.15 | 22.1 | 90 | 10 |
| MC-WR | 1068.6 > 134.9 | 60 | 70 | 0.45 | Phenomenex, Kinetex C_8_ Core Shell, 2.1 x 100 mm, 2.6 µm, 0.3 mL/min flowrate, 10 µL injection | | |
| 7-desmethylated-MC-LR | 981.5 > 134.9 | 75 | 65 | 0.14 |  |  |  |
| MC-HilR | 1009.6 > 134.9 | 70 | 65 | 0.15 |  |  |  |
| 3-desmethylated-MC-LR | 981.5 > 134.9 | 75 | 65 | 0.3 |  |  |  |
| MC-LA | 910.5 > 134.9 | 40 | 50 | 0.15 | **Electrospray Conditions** | | |
| MC-LY | 1002.5 > 134.9 | 40 | 60 | 0.15 | Polarity | | Positive Ion |
| MC-LW | 1025.5 > 134.9 | 45 | 65 | 0.15 | Capillary needle voltage | | +4 kV |
| MC-LF | 986.5 > 134.9 | 40 | 60 | 0.15 | Cone gas flow | | 50 L/h |
| C_2_D_5_-MC-LR (surrogate) | 1028.6 > 134.9 | 55 | 70 |  | N_2_ desolvation gas flow | | 1000 L/h |
| Cyclosporin-A,^13^C_2_, *d*_4_ (IS) | 1208.9 > 99.9 | 65 | 90 |  | Desolvation gas temp. | | 350 ^o^C |

Table S6. Mean and variation (genomic copy numbers mL^-1^) of surface water determined by qPCR targeting 16 rRNA, *rpoC*, *mcyA*, *mcyE* and *mcyG* genes of cyanobacteria (cya), *Microcystis* (mic) and *Plantothrix* (pla)

| *Variable* | N | Mean | Std Dev | Minimum | Maximum |
| --- | --- | --- | --- | --- | --- |
| *Cyanobacteria* | 392 | 2.95×10^6^ | 2.38×10^6^ | 24230 | 1.46×10^7^ |
| *Microcystiis* | 392 | 1.48×10^5^ | 1.95×10^6^ | 0 | 1.22×10^6^ |
| *Planktothrix* | 201 | 1.36×10^5^ | 1.53×10^5^ | 0 | 7.03×10^5^ |
| *Cylindrospermopsis* | 201 | 1.81×10^2^ | 3.61×10^2^ | 0 | 1.60×10^3^ |
| *Nostoc* | 201 | 2.25×10^2^ | 2.31×10^2^ | 0 | 1.03×10^3^ |
| mcyEcya | 392 | 5.63×10^4^ | 5.75×10^4^ | 53 | 3.95×10^5^ |
| mcyAcya | 392 | 4.02×10^4^ | 4.48×10^4^ | 0 | 3.50×10^5^ |
| mcyAmic | 392 | 5.10×10^4^ | 6.75×10^4^ | 0 | 4.85×10^5^ |
| mcyGmic | 392 | 4.39×10^4^ | 5.33×10^4^ | 0 | 4.11×10^5^ |
| mcyEpla | 201 | 4.05×10^2^ | 5.70×10^2^ | 0 | 2.89×10^3^ |

Table S7 Pairwise comparisons of qPCR targeting cyanobacteria 16S rRNA gene (CYA), *Microcystis* 16 rRNA gene (MIC) and microcystin producing gene (mcyEcya) (log gn mL^-1^, df=388)

| **logCYA comparisons significant at the 0.05 level are indicated by ***.** | | | | | **logMIC comparisons significant at the 0.05 level are indicated by ***.** | | | | | **log mcyEcya comparisons significant at the 0.05 level are indicated by ***.** | | | | |
| --- | --- | --- | --- | --- | --- | --- | --- | --- | --- | --- | --- | --- | --- | --- |
| **site** | **Difference** | **Simultaneous 95% Confidence** | |  | **site** | **Difference** | **Simultaneous 95% Confidence** | |  | **site** | **Difference** | **Simultaneous 95% Confidence** | |  |
| **Comparison** | **Between** | **Limits** | |  | **Comparison** | **Between** | **Limits** | |  | **Comparison** | **Between** | **Limits** | |  |
|  | **Means** |  | |  |  | **Means** |  | |  |  | **Means** |  | |  |
| **CGB - BUOY** | 0.039 | -0.2037 | 0.28213 |  | **EFLS - EMB** | 0.04005 | -0.642 | 0.72238 |  | **EMB - BUOY** | 0.00614 | -0.3875 | 0.39976 |  |
| **CGB - EFLS** | 0.044 | -0.1923 | 0.28066 |  | **EFLS - BUOY** | 0.05261 | -0.63 | 0.73495 |  | **EMB - CGB** | 0.00834 | -0.3843 | 0.40093 |  |
| **CGB - EMB** | 0.087 | -0.1557 | 0.33006 |  | **EFLS - CGB** | 0.06823 | -0.612 | 0.74869 |  | **EMB - EFLS** | 0.02679 | -0.3565 | 0.41006 |  |
| **BUOY - CGB** | -0.039 | -0.2821 | 0.20365 |  | **EMB - EFLS** | -0.04005 | -0.722 | 0.64229 |  | **BUOY - EMB** | -0.00614 | -0.3998 | 0.38748 |  |
| **BUOY - EFLS** | 0.005 | -0.2322 | 0.24208 |  | **EMB - BUOY** | 0.01257 | -0.688 | 0.71332 |  | **BUOY - CGB** | 0.0022 | -0.3904 | 0.39479 |  |
| **BUOY - EMB** | 0.048 | -0.1956 | 0.29146 |  | **EMB - CGB** | 0.02819 | -0.671 | 0.72711 |  | **BUOY - EFLS** | 0.02065 | -0.3626 | 0.40392 |  |
| **EFLS - CGB** | -0.044 | -0.2807 | 0.19227 |  | **BUOY - EFLS** | -0.05261 | -0.735 | 0.62972 |  | **CGB - EMB** | -0.00834 | -0.4009 | 0.38426 |  |
| **EFLS - BUOY** | -0.005 | -0.2421 | 0.23216 |  | **BUOY - EMB** | -0.01257 | -0.713 | 0.68819 |  | **CGB - BUOY** | -0.0022 | -0.3948 | 0.3904 |  |
| **EFLS - EMB** | 0.043 | -0.1941 | 0.2801 |  | **BUOY - CGB** | 0.01562 | -0.683 | 0.71455 |  | **CGB - EFLS** | 0.01845 | -0.3638 | 0.40067 |  |
| **EMB - CGB** | -0.087 | -0.3301 | 0.15571 |  | **CGB - EFLS** | -0.06823 | -0.749 | 0.61222 |  | **EFLS - EMB** | -0.02679 | -0.4101 | 0.35649 |  |
| **EMB - BUOY** | -0.048 | -0.2915 | 0.19558 |  | **CGB - EMB** | -0.02819 | -0.727 | 0.67074 |  | **EFLS - BUOY** | -0.02065 | -0.4039 | 0.36263 |  |
| **EMB - EFLS** | -0.043 | -0.2801 | 0.19414 |  | **CGB - BUOY** | -0.01562 | -0.715 | 0.68331 |  | **EFLS - CGB** | -0.01845 | -0.4007 | 0.36377 |  |

Table S8. Comparisons of monthly quantities (genome copy numbers mL^-1^) of qPCR targeting 16 rRNA, *rpoC*, *mcyA*, *mcyE* and *mcyG* genes of cyanobacteria (cya), *Microcystis* (mic) and *Plantothrix* (pla) using the t-test of Least Significant Difference, Tukey's Studentized Range, Bonferroni and Scheffe's Test based on normalized data using log transformation for all pairwise comparisons at the 0.05 level indicated by ***

|  | May |  |  | June |  |  | July |  |  | August | |  | |  | | September | | |  | Comparisons |
| --- | --- | --- | --- | --- | --- | --- | --- | --- | --- | --- | --- | --- | --- | --- | --- | --- | --- | --- | --- | --- |
| **Variable** | N | Mean | Std Dev | N | Mean | Std Dev | N | Mean | Std Dev | N | Mean | | Std Dev | | N | | Mean | Std Dev | | *** |
| **CYA** | 44 | 2.87×10^5^ | 3.70×10^5^ | 180 | 4.06×10^6^ | 2.53×10^5^ | 80 | 3.73×10^6^ | 1.39×10^6^ | 40 | 2.03×10^6^ | | 8.90×10^5^ | | 40 | | 7.64×10^5^ | 6.00×10^5^ | | 5-6, 5-7, 5-8, 5-9, 6-7, 6-8,6-9, 7-8, 7-9, 8-9 |
| **MIC** | 44 | 8.04×10^2^ | 2.24×10^3^ | 180 | 1.29×10^5^ | 1.37×10^5^ | 80 | 4.06×10^5^ | 2.15×10^5^ | 40 | 590×10^4^ | | 5.92×10^4^ | | 40 | | 1.18×10^3^ | 8.60×10^2^ | | 5-6, 5-7, 5-8, 6-7,6-8,6-9, 7-8, 7-9 |
| **mcyEcya** | 44 | 1.59×10^3^ | 2.74×10^3^ | 180 | 5.30×10^4^ | 3.62×10^4^ | 80 | 1.30×10^5^ | 5.92×10^4^ | 40 | 4.77×10^4^ | | 4.27×10^4^ | | 40 | | 3.37×10^3^ | 4.57×10^3^ | | 5-6, 5-7, 5-8, 5-9, 6-7, 6-9, 7-8, 7-9, 8-9 |
| **mcyAcya** | 44 | 5.76×10^2^ | 9.67×10^2^ | 180 | 4.18×10^4^ | 350×10^4^ | 80 | 9.25×10^4^ | 4.74×10^4^ | 40 | 1.85×10^4^ | | 1.60×10^4^ | | 40 | | 1.65×10^3^ | 8.00×10^2^ | | 5-6, 5-7, 6-7, 6-8, 6-9, 7-8, 7-9 |
| **mcyAmic** | 44 | 1.77×10^2^ | 4.96×10^2^ | 180 | 4.29×10^4^ | .00×10^4^ | 80 | 1.42×10^5^ | 8.22×10^4^ | 40 | 2.23×10^4^ | | 2.24×10^4^ | | 40 | | 5.25×10^2^ | 3.83×10^2^ | | 5-6, 5-7, 6-7, 6-9, 7-8, 7-9 |
| **mcyGmic** | 44 | 1.98×10^2^ | 5.51×10^2^ | 180 | 4.14×10^4^ | 3.59×10^4^ | 80 | 1.12×10^5^ | 6.13×10^4^ | 40 | 1.94×10^4^ | | 1.86×10^4^ | | 40 | | 4.04×10^2^ | 2.68×10^2^ | | 5-6, 5-7, 6-7, 6-8, 6-9, 7-8, 7-9 |
| **PLA** | 22 | 3.39×10^3^ | 5.70×10^3^ | 91 | 1.24×10^5^ | 7.26×10^4^ | 44 | 3.22×10^5^ | 2.03×10^5^ | 20 | 4.80×10^4^ | | 3.15×10^4^ | | 20 | | 1.33×10^4^ | 7.20×10^3^ | | 5-6, 5-7, 6-7, 6-8, 6-9, 7-8, 7-9, |
| **mcyEpla** | 22 | 2 | 3 | 91 | 4.21×10^2^ | 6.27×10^2^ | 44 | 7.36×10^2^ | 6.43×10^2^ | 20 | 2.67×10^2^ | | 1.11×10^2^ | | 20 | | 1.77×10^2^ | 9.77×10^1^ | | 5-6, 5-7, 6-7, 7-8, 7-9, |
